# Amphiphilic Polyphosphonate Copolymers as New Additives for PDMS-Based Antifouling Coatings

**DOI:** 10.3390/polym13193414

**Published:** 2021-10-05

**Authors:** Elisa Guazzelli, Niccolò Lusiani, Gianfranca Monni, Matteo Oliva, Chiara Pelosi, Frederik R. Wurm, Carlo Pretti, Elisa Martinelli

**Affiliations:** 1 Dipartimento di Chimica e Chimica Industriale, Università di Pisa, 56124 Pisa, Italy; elisa.guazzelli@dcci.unipi.it (E.G.); n.lusiani@studenti.unipi.it (N.L.); chiara.pelosi92@gmail.com (C.P.); 2 Dipartimento di Scienze Veterinarie, Università di Pisa, 56126 Pisa, Italy; gianfranca.monni@unipi.it (G.M.); carlo.pretti@unipi.it (C.P.); 3 Consorzio Interuniversitario di Biologia Marina e Ecologia Applicata ‘‘G. Bacci’’, 57128 Livorno, Italy; oliva@cibm.it; 4 Sustainable Polymer Chemistry, Department of Molecules and Materials, MESA+ Institute for Nanotechnology, Faculty of Science and Technology, Universiteit Twente, P.O. Box 217, 7500 AE Enschede, The Netherlands

**Keywords:** polyphosphoester, amphiphilic polymer, polysiloxane, polyethylene glycol, antifouling, fouling release

## Abstract

Poly(ethyl ethylene phosphonate)-based methacrylic copolymers containing polysiloxane methacrylate (SiMA) co-units are proposed as surface-active additives as alternative solutions to the more investigated polyzwitterionic and polyethylene glycol counterparts for the fabrication of novel PDMS-based coatings for marine antifouling applications. In particular, the same hydrophobic SiMA macromonomer was copolymerized with a methacrylate carrying a poly(ethyl ethylene phosphonate) (PEtEPMA), a phosphorylcholine (MPC), and a poly(ethylene glycol) (PEGMA) side chain to obtain non-water soluble copolymers with similar mole content of the different hydrophilic units. The hydrolysis of poly(ethyl ethylene phosphonate)-based polymers was also studied in conditions similar to those of the marine environment to investigate their potential as erodible films. Copolymers of the three classes were blended into a condensation cure PDMS matrix in two different loadings (10 and 20 wt%) to prepare the top-coat of three-layer films to be subjected to wettability analysis and bioassays with marine model organisms. Water contact angle measurements showed that all of the films underwent surface reconstruction upon prolonged immersion in water, becoming much more hydrophilic. Interestingly, the extent of surface modification appeared to be affected by the type of hydrophilic units, showing a tendency to increase according to the order PEGMA < MPC < PEtEPMA. Biological tests showed that *Ficopomatus enigmaticus* release was maximized on the most hydrophilic film containing 10 wt% of the PEtEP-based copolymer. Moreover, coatings with a 10 wt% loading of the copolymer performed better than those containing 20 wt% for the removal of both *Ficopomatus* and *Navicula*, independent from the copolymer nature.

## 1. Introduction

Polyphosphoesters (PPEs) are an innovative class of phosphorus-based polymers that offer a platform for biodegradable and biocompatible macromolecules. In particular, main-chain PPEs are characterized by repeating phosphoester bonds in the backbone, and the pentavalent phosphorus atom can be exploited to graft different side chains, thus providing a useful tool for tuning the polymer properties [1]. Among the others, polyphosphonates are a subset of PPEs bearing an alkyl/aryl group in the side chain, linked to the phosphorus atom through a phosphonate bond [2]. The hydrolysis rate can be tuned by modifying the chemical structure of the lateral substituent, e.g., the longer and sterically bulkier the side chain is, the better it shields the main chain from nucleophilic attack [3]. Moreover, the substitution of the side-group chain changes the water solubility of polyphosphonates, e.g., the polymer becomes insoluble in water as well as partly crystalline going from isopropyl to hexyl group [4,5,6]. Tunable crystallinity, along with hydrophilicity, thermo-responsiveness, biocompatibility, potential biodegradability, possibility to introduce a functional group in the chain end, and similarity to biomacromolecules such as nucleic acids, are some of the many aspects that make polyphosphonates very appealing for biological and biomedical applications [7]. They have been studied as drug nanocarriers [8], or in protein-polymer conjugation [9].

Nonetheless, only in recent years has there been a surge in interest in this class of polymers that still represent a young field of study. Anionic ring opening polymerization (AROP) [10,11] and other polymerization methods [7,12] have proved to afford polyphosphonates with a remarkable control over molecular weight, dispersity, and architecture. This class of hydrophilic polymers could offer an attractive alternative to polyethylene glycol (PEG) and polyzwitterions, as PPEs are not plagued by uncontrolled oxidative degradation. In particular, aiming to intrinsically benign, biocide-free antifouling (AF) and fouling-release (FR) surfaces, in the last years much effort was devoted to the design of coatings with optimal performance achieved through several approaches, centered on tuning morphology, topography, surface reconstruction, segregation, and wettability of polymer films [13,14,15,16,17,18,19,20,21,22]. Particularly promising, the FR approach relies on the combination of low surface energy, low elastic modulus, and low surface roughness typical of polydimethylsiloxanes [23,24,25,26], facilitating the release of the fouling biomass in presence of relatively low hydrodynamic forces, e.g., the shear stress induced on ship hulls during navigation. Nevertheless, FR siloxane coatings require improvements to overcome some disadvantages, including the higher fouling accumulation during idle time [26] and the low fouling release efficacy towards some microalgae diatoms [27]. As a result, hydrophilic fouling-resistant polymers, including PEG and polyzwitterions, were extensively investigated for the hydrophilization of hydrophobic PDMS-based FR coatings [28,29,30,31,32,33,34,35]. PEG, especially, is considered a “gold standard” in the field of anti-biofouling coatings, even though it was proved to undergo oxidative degradation generating some toxic compounds, including 1,4-dioxane and formaldehyde [36]. Unlike PEG, polyzwitterions show significant stability to degradation even in relatively aggressive environments such as sea water, making them suitable for long-term applications in the marine industry [37]. Nevertheless, they suffer from several disadvantages, including high hygroscopicity and cost as well as poor solubility in most commonly employed solvents [38]. Thus, herein, we focused on finding a new class of water-soluble and eco-sustainable polymers that could overcome such issues [1,36]. Among others, we tested PPEs as an emerging and attractive class of materials for the substitution of PEG in the field of non-toxic, anti-biofouling coatings. A comparative study of protein adsorption on model monolayer surfaces composed of polyphosphates and polyphosphonates has been reported in the literature, demonstrating that protein resistance can be achieved, and antifouling properties are strongly affected by the nature of polyphosphoester side chains [39]. Except for this study, PPEs’ antifouling potential remains largely unexplored to date. To the best of our knowledge, nothing is reported in the literature about the use of polyphosphonates to combat marine fouling. For this specific application, the investigation of the hydrolysis kinetics of polyphosphonate-based polymers in seawater conditions is of main significance for their possible exploitation as hydrolysable self-polishing coatings capable of generating a dynamic, water-responsive, and evolving surface unsuitable for the settlement of marine organisms. Hydrolyzable polymers are traditionally used for the development of biocide-based erodible coatings with a self-polishing (SP) effect [14]. New strategies recently emerged in the field, which combine the advantages of biocide-free SP/FR in hybrid systems, incorporating hydrolyzable trialkylsilyl or bis(trimethylsiloxy)methylsilyl methacrylate-based polymers into a polymer matrix, either as an additive [40,41] or as a structural component [42,43,44]. When the polymer hydrolyzes, the surface becomes more hydrophilic and responsive to the external environment. The eventual dissolution in water of the hydrolyzed components thus generates a new fresh coating layer, according to a self-polishing mechanism. Such an evolving surface was shown to be generally beneficial in enhancing AF performance against model macrofoulers and in real seawater environments [15,45].

With these rationales in mind, new amphiphilic random copolymers derived from a hydrophilic poly(ethyl ethylene phosphonate) methacrylate (PEtEPMA) and a commercially available hydrophobic polydimethylsiloxane methacrylate (SiMA) were synthesized via free radical polymerization with the aim to investigate the anti-biofouling potential of polyphosphonate-based copolymers toward marine organisms. SiMA was also alternatively copolymerized with the zwitterionic phosphorus-based monomer, 2-methacryloxyethyl phosphorylcholine (MPC), and poly(ethylene glycol) methyl ether methacrylate (PEGMA) taken as reference samples, due to the fact that their antifouling properties were well-established [15]. PEtEPMA-, MPC-, and PEGMA-based copolymers were used as surface-active additives to prepare condensation cure PDMS-based coatings for surface and biological evaluations. In particular, two different organisms, *viz* the serpulid *Ficopomatus enigmaticus* and the diatom *Navicula salinicola* were selected to study the antifouling and fouling release potential of the prepared amphiphilic coatings.

## 2. Materials and Methods

### 2.1. Materials

Polydimethylsiloxane mono methacryloxy-propyl-terminated (SiMA, Fluorochem: 10 cSt, *M*_n_ = 1000 g/mol) and poly(ethylene glycol) methyl ether methacrylate (PEGMA, Sigma Aldrich, *M*_n_ = 300 g/mol) were dissolved in chloroform and filtered on basic alumina to remove the polymerization inhibitors. Ethanol (EtOH, absolute Carlo Erba) was stored under nitrogen, with over 4 Å molecular sieves. Tetrahydrofuran (THF, Carlo Erba) and toluene (Sigma-Aldrich) were refluxed over calcium hydride for 4 h and then distilled at atmospheric pressure under nitrogen. Moreover, 2-2′-azobisisobutyronitrile (AIBN, Fluka) was cold and recrystallized from methanol. Tetra-*n*-butylammonium fluoride trihydrate (TBAF, Merk), poly(diethoxysiloxane) (ES40, ABCR, *M*_n_ = 134 g/mol), 2-methacryloyloxyethyl phosphorylcholine (MPC, Sigma-Aldrich), and poly(dimethylsiloxane) bis silanol-terminated (HO-PDMS-OH, ABCR, *M*_n_ = 26,000 g/mol, *Ð* = 1.45) were used as received. Furthermore, 2-(Benzyloxy)-ethanol was purchased from ABCR, distilled from calcium hydride, and stored over molecular sieve (4 Å) and under argon prior to use. The 8-diazabicyclo(5.4.0)undec-7-ene (DBU) was purchased from Sigma-Aldrich, distilled prior to use, and stored over molecular sieve (4 Å) under argon. The 2-Isocyanatoethyl methacrylate was purchased from Sigma-Aldrich (Germany). Dry solvents were purchased from Across Organic (Germany). Other common laboratory reagents and solvents were used as received.

### 2.2. Synthesis

#### 2.2.1. PEtEPMA Synthesis

The monomer 2-ethyl-2-oxo-1,3,2-dioxaphospholane (EtEP) was polymerized by AROP using 2-(benzyloxy)-ethanol and 1,8-diazabicyclo(5.4.0)undec-7-ene (DBU) as initiator and catalyst, respectively. EtEP was synthesized according to a literature procedure [6].

EtEP (5.47 g, 18 eq, 40.21 mmol) was placed in a flame-dried Schlenk tube, dissolved in dry benzene, and dried by repeated lyophilization. It was dissolved in dry dichloromethane to reach a concentration of 4 M. A solution of initiator (339.4 mg, 1 eq, 2.23 mmol) in 10 mL of dry dichloromethane was prepared and added to the monomer solution via gastight syringe (Hamilton). The monomer solution and the DBU were adjusted to 25 °C. The polymerization was initiated by the rapid addition of DBU (1.02 g, 3 eq, 6.70 mmol) to the monomer solution. The polymerization was quenched after 16 h by the rapid addition of an excess of 2-isocyanatoethyl methacrylate (1.59 g, 4.60 eq, 10.28 mmol, 1.44 mL). The macromonomer was purified by repeated precipitation from dichloromethane solutions into ice-cold diethyl ether (−60 °C) (yield 75%; *M*_n_ = 1140 g/mol and *Ð* = 1.44).

^1^H NMR (DMSO-*d*_6_ ppm): δ 7.48–7.29 (m, aromatic protons), 6.12 (s, cis H-CH_2_=CH_2_ end group) 5.78 (s, trans H-CH_2_=CH_2_ end group), 4.53 (s, aryl-CH_2_-), 4.48–4.01 (m, backbone -CH_2_-CH_2_-), 3.3–3.4 (m, CH_2_-N), 2.07–1.52 (m, side-chain -P-CH_2_- + H-CH_2_-CH_2_=CH_2_ end group), 1.27–0.82 (m, side-chain -CH_3_). ^31^P NMR (DMSO-*d*_6_, ppm): δ 34.63 (backbone), 34.38 (terminal).

#### 2.2.2. SiMA-*co*-PEtEPMAx Copolymer Synthesis

In a typical polymerization, SiMA (0.240 g, 0.240 mmol), PEtEPMA (0.760 g, 0.240 mmol), AIBN (10.0 mg, 0.0609 mmol), ethanol (0.3 mL), and toluene (0.6 mL) were loaded in a 10 mL Carius tube, and the reaction mixture was degassed by three freeze-pump-thaw cycles. The polymerization was carried out under vacuum at 70 °C in an oil bath under magnetic stirring for 24 h. The reaction was stopped by exposure to air and cooled down to room temperature. A ^1^H NMR spectrum of a sample of the crude product was acquired to assess monomer conversion (*p* = 96%). The polymer was purified by precipitations into a large excess of hexane from chloroform solutions (yield 54%). The obtained copolymer, named SiMA-*co*-PEtEPMA53, contained 53 mol% of PEtEPMA co-units.

^1^H NMR (400 MHz, chloroform-d): δ (ppm) = 7.40–7.33 (OCH_2_**C_6_H_5_**); 4.58 (O**CH_2_**C_6_H_5_); 4.40–4.00 (OCH_2_CH_2_O, COOCH_2_); 3.70 (CH_2_**CH_2_**NH); 3.50 (DBU) 2.00–1.50 (SiCH_2_**CH_2_CH_2_**CH_3_, COOCH_2_**CH_2_**CH_2_, **CH_2_**CCH_3_, P**CH_2_**CH_3_); 1.50–0.63 (CH_2_C**CH_3_**, PCH_2_**CH_3_**, SiCH_2_CH_2_CH_2_**CH_3_**); 0.55 (Si**CH_2_**CH_2_); 0.08 (Si**CH_3_**).

The same purification procedure was adopted for SiMA-*co*-PEtEPMA66, while SiMA-*co*-PEtEPMA20 was purified by repeated precipitations from dichloromethane solutions into a large excess of a mixture of water and methanol (50% vol).

Homopolymer p(MPC) and p(SiMA) were also prepared by conventional radical polymerization as reference samples.

#### 2.2.3. SiMA-*co*-MPC20 and SiMA-*co*-PEGMA28 Copolymer Synthesis

SiMA-*co*-MPC20 was prepared as follows. SiMA (1.79 g, 1.79 mmol), MPC (0.22 g, 0.77 mmol), AIBN (20.1 mg, 0.122 mmol), THF (2.5 mL), and ethanol (2.5 mL) were loaded in a 25 mL Carius tube, and the reaction mixture was degassed by three freeze-pump-thaw cycles. After the last cycle, the tube was backfilled with nitrogen, and polymerization was carried out at 70 °C in an oil bath under magnetic stirring for 24 h. The reaction was stopped by exposure to air and cooled down to room temperature. A ^1^H NMR spectrum of a sample of the crude product was acquired to assess the monomer conversion (*p* = 96%). The polymer was purified by repeated precipitations from chloroform solutions into a large excess of methanol (yield 49%). The obtained copolymer (*M*_n_ = 11700 g/mol, *Ð* = 1.4), named SiMA-*co*-MPC20, contained 20 mol% of MPC units.

^1^H NMR (400 MHz, chloroform-d/methanol-d_4_ = 2/1 *v*/*v*): δ (ppm) = 4.20–3.60 (COOCH_2_**,** CH_2_O(PO_2_-)OCH_2_, CH_2_O(PO_2_-)O**CH_2_**); 3.52 (**CH_2_**N^+^(CH_3_)_3_); 3.07 (CH_2_N^+^(**CH_3_**)_3_); 2.00–0.60 (CH_2_CCH_3_, SiCH_2_**CH_2_CH_2_CH_3_**, COOCH_2_**CH_2_**CH_2_); 0.34 (Si**CH_2_**CH_2_); −0.15 (SiCH_3_).

Homopolymer p(MPC) was also prepared by conventional radical polymerization as a reference sample.

SiMA-*co*-PEGMA28 was prepared as follows. SiMA (1.82 g, 1.82 mmol), PEGMA (0.190 g, 0.633 mmol), AIBN (15.0 mg, 0.0914 mmol), and toluene (4.9 mL) were loaded in a 25 mL Carius tube, and the reaction mixture was degassed by three freeze-pump-thaw cycles. The polymerization was carried out under vacuum at 70 °C in an oil bath under magnetic stirring for 24 h. The reaction was stopped by exposure to air and cooled down to room temperature. A ^1^H NMR spectrum of a sample of the crude product was acquired to assess the monomer conversion (*p* = 85%). The polymer was purified by repeated precipitations from dichloromethane solutions into a large excess of methanol (yield 62%). The obtained copolymer (*M*_n_ = 46200 g/mol, *Ð* = 3.5), named SiMA-*co*-PEGMA28, contained 28 mol% of PEGMA units.

^1^H NMR (400 MHz, dichloromethane-d2): δ (ppm) = 4.18 (COOCH_2_, PEGMA); 3.90 (COOCH_2_, SiMA) 3.77–3.50 (OCH_2_CH_2_O); 3.38 (OCH_3_) 2.3–0.80 (SiCH_2_**CH_2_CH_2_**CH_3_, COOCH_2_**CH_2_**CH_2_, **CH_2_**CCH_3_CH_2_C**CH_3_**, SiCH_2_CH_2_CH_2_**CH_3_**); 0.59 (Si**CH_2_**CH_2_); 0.12 (SiCH_3_).

### 2.3. Film Preparation

#### 2.3.1. Three-Layer PDMS-Based Polymer Films

**Figure 1:**
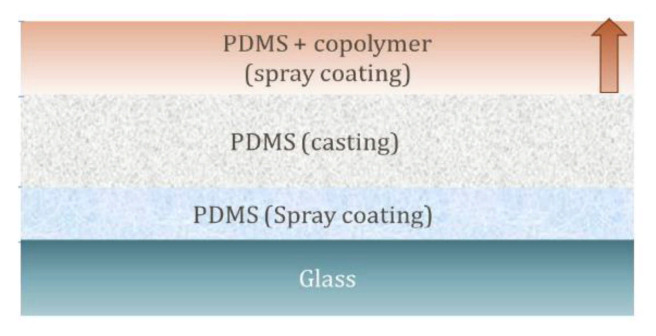
Schematic representation of three-layer PDMS-based films.

Films were deposited on clean glass slides (76 × 26 mm^2^). Films were prepared according to a three-step procedure, with the resulting geometry reproduced in Figure 1.

The first step consisted of spray-coating the following ethyl acetate solution onto the glass slides using a Badger model 250 airbrush. A solution containing the bis silanol-terminated PDMS matrix (1 g), the crosslinker ES40 (25 mg) and the catalyst/activator TBAF (0.8 mg) that was dissolved in ethyl acetate (3 mL) was sprayed on each glass slide (dry thickness < 5 μm). Approximatively 0.5 mL of solution were sprayed on each glass slide.

A solution of the same composition was pre-sonicated for 10 min and then cast onto the bottom layer (~400 μm thickness). Subsequently, a final top layer was deposited by spray-coating the PDMS-based formulation reported above, in which the copolymer additive (10 wt% or 20 wt% relative to HO-PDMS-OH) was added. Approximately 0.5 mL of solution were sprayed on each glass slide. After the deposition of each layer, the films were left at room temperature for 12 h and then thermally annealed in a vacuum oven at 120 °C for 12 h.

#### 2.3.2. Single Layer Polymer Films

Single layer films were deposited on clean glass slides (18 × 18 mm^2^) by spin-coating polymer solutions. In the case of the homopolymer p(PEtEPMA) pristine films, glass surface activation was needed to avoid de-wetting. The activation was performed in a plasma chamber under an oxygen atmosphere (Pico PCCE device by Diener electronic Gmbh and Co. KG, Ebhausen, Germany) set with the following parameters: pressure of 0.50 mbar, power of 200 W, and duration of 60 min.

Approximately 100 μL of the polymeric solution was deposited on each glass slide, and the film was spin-coated with a WS-400B-6NPP-LITE (Laurell Technologies Corp., North Wales, PA, USA) spin-coater operating at 5000 rpm for 30 s. Pristine polymer films were produced from 30 mg/mL solutions in ethanol, except for SiMA-*co*-MPC20 and SiMA-*co*-PEGMA28, which were prepared by using ethyl acetate as solvent. On the other hand, PDMS-based films were fabricated with the same formulations used for the topmost layer in the three-layer polymer films, with 10 wt% or 20 wt% copolymer additives with respect to HO-PDMS-OH. After the deposition, the films were left at room temperature for 3 h and then annealed in a vacuum oven at 120 °C for 12 h.

### 2.4. Characterization

The ^1^H and ^31^P NMR measurements were carried out on a Bruker Avance 400 MHz spectrometer with deuterated solvents at room temperature. The sample concentration was approximately 20–25 g/L. For ^31^P NMR, chemical shifts were referred to as H_3_PO_4_, to which was attributed a shift of 0 ppm in D_2_O. For the ^1^H NMR spectra, the internal standard was the solvent peak.

The hydrolysis of the phosphonate was monitored by ^31^P NMR. The samples of the homopolymers and of the macromonomer PEtEP (10 mg) were dissolved in 800 μL of a mixture 50/50 *v*/*v* of D_2_O and artificial sea water (pH = 8.2) or bicarbonate buffer solution (pH = 10). In the case of water-insoluble copolymers, SiMA-*co*-MPC20 and SiMA-*co*-PEtEPMA20 (10 mg) were dissolved in 1 mL of THF-*d*_8_, to which 100 μL of sodium bicarbonate buffer solution was added. The ^31^P NMR spectra were recorded at different time intervals.

The number and weight average molecular weights (*M*_n_, *M*_w_) were determined by gel permeation chromatography (GPC), using a Jasco PU-2089Plus liquid chromatograph pump equipped with two PL gel 5 μm mixed-D columns (200–400,000 g/mol), a Jasco RI-2031 Plus refractive index detector, and a Jasco UV-2077Plus UV/vis detector. Measurements were carried out using chloroform (or THF in the case of SiMA-*co*-MPC20) as the mobile phase, at a flux of 1 mL/min and a temperature of 30 °C maintained by a Jasco CO 2063 Plus column thermostat. Polymethyl methacrylate (PMMA) standards were used for calibration. Samples were prepared at the concentration levels of 5 mg/mL and 1 mg/mL, and they were filtered through a 0.2 μm PTFE filter before injection.

Differential scanning calorimetry (DSC) analyses were performed with a Mettler DSC 922e instrument. Approximately 10 mg of polymer were analyzed in 40μL aluminum standard crucibles. The glass transition temperatures were taken as the inflection temperatures in the second heating ramp (from −140 °C to 120 °C at 10 °C/min) obtained with STARe software (v. 9.01).

Static contact angle was determined using the sessile drop method with a Camtel FTA200 Drop Shape Analyzer with FTA 32 software, using water of the highest purity available as testing liquid.

#### 2.4.1. Biological Tests

All films for laboratory assays were immersed in artificial seawater for 48 h prior to tests.

##### *Navicula salinicola* Settlement and Detachment Assay

The settlement of the diatom *N. salinicola* was assessed by exposing the films to a 10^4^ cells/mL algal suspension in static conditions (temperature 22 °C; photoperiod 14:10 h light:darkness; light intensity 3000 lux), for 24 h. After this period, all films were gently rinsed with clean seawater, and the adherent algae were evaluated by measuring the autofluorescence of chlorophyll (a+b) with a microplate reader (Synergy-HTX, Biotek, Winooski, VT, USA). Briefly, before exposure to the algal solution, the intrinsic fluorescence emission (at 435 nm and 682 nm) of the coated slides was measured as background noise. The calibration of biomass, in terms of chlorophyll (a+b) content, was performed measuring the fluorescence intensity of several known algal concentrations at 435 nm and 682 nm, and calculating a regression line equation. The homogeneous distribution of diatoms on film surfaces was evaluated in optical microscopy. After the algal adherent biomass evaluation, the coated slides were subjected to a detachment assay. Briefly, slides were exposed for 5 min to a 5 Pa shear stress (corresponding to a 50 L/min water stream), using a turbulent channel flow apparatus. After this step, still adherent algal biomass was measured again, as described above. Then, the percentage of detached algal biomass was calculated.

##### Settlement of Larvae and Adult Detachment of *Ficopomatus enigmaticus*

Adults of *F. enigmaticus* were collected in S. Rossore–Migliarino–Massaciuccoli Regional Park (Pisa, Italy) and acclimated in a laboratory to test conditions (temperature 20 ± 2 °C, water salinity 30, pH 8.12, photoperiod 14:10 h light:darkness) prior to use. Larval culture for test setup was produced as follows: male and female gamete release was obtained by gently breaking serpulids’ calcareous tubes, removing worms, and placing them singularly in 24-well plates filled with 0.45 μm of filtered seawater (salinity 30). After gametes were released, sperms of different males were pooled together, and the final concentration was measured with a Burker cell-counting chamber and an optical microscope. In the meantime, eggs of different females were pooled together and rinsed 3 times with clean 0.45 μm filtered seawater, in order to remove broken and immature eggs. A fertilization suspension (~800 mL) was then prepared, exposing all of the eggs to a sperm concentration of about 3–4 × 10^5^ sperms/mL. Fertilization usually occurred within 1 h, while first swimming larval development occurred within 18–24 h. The needed larval stage for settlement assay was the “competent larva”, i.e., a larva that loses its swimming ability, starts to crawl on hard substrata, and actively searches for settlement spots. This stage is usually reached after 9–10 days from fertilization. For this reason, larvae were reared until they reached “competent larva” stage. Rearing conditions were the same as for the adult maintenance. When the wanted stage was reached, larvae were collected with a 30 μm nylon net and resuspended in a small volume of fresh seawater (~50 mL). Simultaneously, coated slides for testing were placed in 4-well Quadriperm^®^ plates, and 1 mL drops of clean seawater were put on each replicate (6 replicates per formulation).

Moreover, 20 competent larvae were pipetted in each drop. All plates were then incubated in darkness at 21 ± 2 °C, covered with wet towels to prevent evaporation. Incubation was performed for a total time of 5 days, counting settled larvae at the end of the exposure and calculating the settlement percentage on each sample replica. PDMS covered slides acted as the negative control.

After the 5-day settlement stages, new competent larvae that were reared in a culture prepared ad hoc were added on each slide (approximately 50 larvae per slide) in order to increase the number of adhered organisms. At this step, Quadriperm^®^ plate wells were completely filled with 5 mL of fresh 0.45 μm filtered seawater. Moreover, 1 mL of an *Isochrysis galbana* algal culture (~3 × 10^4^ cells/mL) was added in each well as a food source. Starting from the 5th day after the new larval inoculum, water and algal food were renewed 3 times per week. *F. enigmaticus* adherent individuals were reared for 40–45 days, at the same conditions reported before, in order to obtain adults with a tube length > 5 mm.

After the rearing period, adherent individuals of *F. enigmaticus* were counted on each slide; then, slides were exposed to a 25 Pa shear stress (corresponding to 150 L/min), for 15 min, using a turbulent channel flow apparatus. After the exposure, still adherent individuals were counted for each slide, and the mean percentage of detachment was calculated.

##### Statistical Analysis

All biological assay results were statistically analyzed with GraphPad Prism^®^ V.5.01 Software. Briefly, an ANOVA analysis was performed for each test output, followed by a Tukey post-test for multiple comparison. PDMS acted as the negative control for each assay.

## 3. Results and Discussion

### 3.1. PEtEPMA Macromonomer Synthesis

**Scheme 1:**
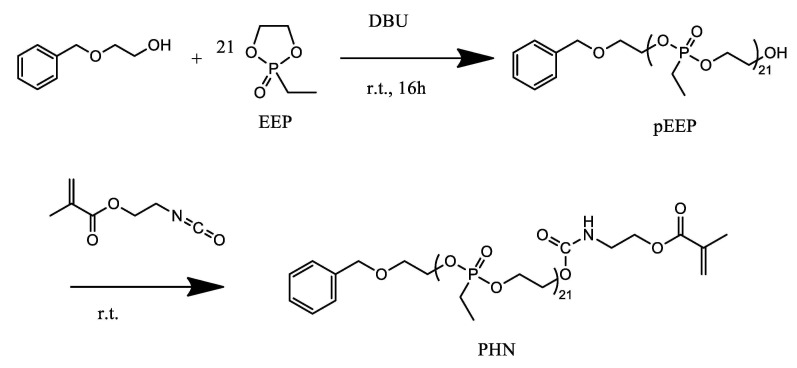
Synthesis of the PEtEPMA macromonomer.

The cyclic monomer 2-ethyl-2-oxo-1,3,2-dioxaphospholane (EtEP) was polymerized by anionic ring opening polymerization (AROP) by using 2-(benzyloxy)-ethanol as the initiator and 1,8-diazabicyclo(5.4.0)undec-7-ene (DBU) as the catalyst. The macromonomer PEtEPMA was obtained by terminating the polymerization with an excess of 2-isocyanatoethyl methacrylate (Scheme 1). Successful polymerization and activation of the chain end was confirmed by ^1^H and ^31^P NMR spectroscopy and by GPC analysis. The average number degree of polymerization of the polyphosphonate side chain was 21, as evaluated by ^1^H NMR in accordance with the previously reported procedures [46,47].

### 3.2. SiMA-co-PEtEPMAx Copolymerization and Chemical-Physical Characterization

**Scheme 2:**
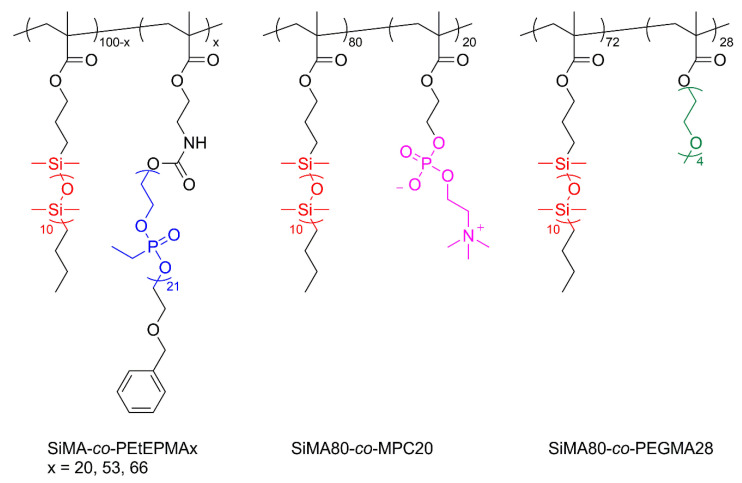
Chemical structure of SiMA-*co*-PEtEPMAx, SiMA-*co*-MPC20, and SiMA-*co*-PEGMA28 amphiphilic copolymers.

Free radical polymerization was chosen as the polymerization technique for the synthesis of copolymers based on SiMA and PEtEPMA macromonomers (Scheme 2). Furthermore, 2,2′-Azobis(2-methylpropionitrile) (AIBN) was chosen as the thermal initiator, and its concentration was kept constant at 1 wt% relative to the comonomers. All copolymerizations were carried out at 70 °C for 24 h in a mixture of ethanol and toluene, 1/2 *v*/*v*, with an initial concentration of comonomers SiMA and PEtEPMA of 0.5 M. p(PEtEPMA) and p(SiMA) homopolymers were also synthesized as a reference sample. A range of comonomer molar ratios were used in the feed in order to prepare copolymers with different molar compositions (Table 1). The obtained polymers were identified as SiMA-*co*-PEtEPMAx, where x is the molar percentage of PEtEPMA units in the copolymer. The molar composition x was calculated from the ratio of the integrated area of the signal at 0.1 ppm and 4.6 ppm, attributed to SiCH_3_ (SiMA) and O**CH_2_**C_6_H_5_ (PEtEPMA), respectively. High conversions (*p* ≥ 94%) were observed for the polymers; nonetheless, the yields were significantly lower due to the difficulty to find a selective non-solvent for the quantitative precipitation of the amphiphilic copolymers. While p(SiMA) was purified by repeated precipitations in methanol, p(PEtEPMA) purification was performed by dialysis in methanol.

### 3.3. SiMA-co-MPC20 and SiMA-co-PEGMA28 Copolymerizations

Two copolymers, namely SiMA-*co*-MPC20 and SiMA-*co*-PEGMA28, were also prepared as reference samples for the phosphonate-based counterpart. The copolymerizations were carried out at 70 °C for 24 h in ethanol and THF (1/1 *v*/*v*) in the case of SiMA-*co*-MPC20, or toluene in the case of SiMA-*co*-PEGMA28. AIBN was used as the thermal initiator, starting from an initial concentration of comonomers of 0.5 M. Copolymers were purified by repeated precipitations from chloroform solutions into methanol. The molar composition of copolymers (Table 1) was calculated from the integrated areas of characteristic ^1^H NMR peaks at 0.1 ppm attributed to SiCH_3_ (SiMA), 3.4 ppm for OCH_3_ (PEGMA), or 3.1 ppm for CH_2_N^+^(**CH_3_**)_3_ (MPC).

### 3.4. Thermal Analysis of the Copolymers

**Figure 2:**
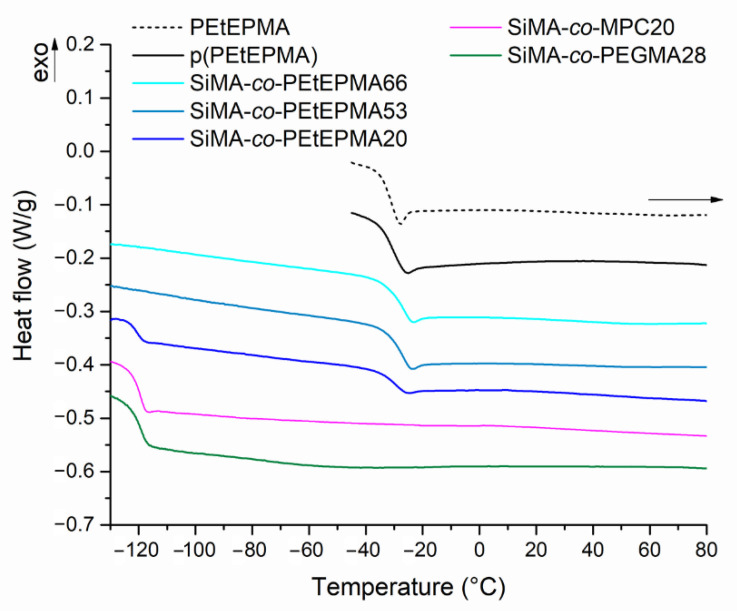
DSC second heating curves of the amphiphilic copolymers.

The thermal properties of polymers were investigated by differential scanning calorimetry (DSC, Figure 2).

Polymers were studied by DSC between −140 and 120 °C, at 10 °C/min in heating and cooling scans. After the first heating, samples were kept at 120 °C for 15 min to remove most of the humidity absorbed, since water would act as a plasticizer, thus lowering the *T*_g_ of the samples. This was especially true for highly hygroscopic polymers, like those carrying zwitterionic and phosphonate side groups.

All of the polymers were found to be completely amorphous (Table 2). Poly(alkyl ethylene phosphonate)s with short alkyl side chains are reported to have *T*_g_s around −40/−45 °C, and in particular, poly(ethyl ethylene phosphonate)s with various molecular weights exhibit *T*_g_ values lower than −50 °C [2,48]. PEtEPMA and p(PEtEPMA) showed glass transition temperatures at ca. −31 °C. SiMA-*co*-PEtEP20 presented two *T*_g_s at −121 °C and −29 °C, attributed to the SiMA and PEtEPMA components, respectively. In contrast, copolymers with a higher ratio of PEtEPMA (above ≥ 53 mol%) only showed a single *T*_g_, close to the value of the polyphosphonate homopolymers. The presence of only one *T*_g_ in the thermogram was likely due to the relatively low weight amount of SiMA in the copolymers.

SiMA-*co*-PEGMA28 exhibited only the SiMA related transition at −119 °C. Since the PEGMA homopolymer with the same length of the oxyethylenic side chain is reported to have a *T*_g_ of −54 °C [49], its absence in the thermogram was ascribed to the low weight fraction of PEGMA in the copolymer. Copolymer SiMA-*co*-MPC20 did not present any other glass transition temperatures, other than the one at −119 °C due to the SiMA units. As reported in the literature, dry polyzwitterions do not exhibit a glass transition temperature within the limits of their thermal stability, as the strong electrostatic intermolecular interactions drastically restrict the chain motion [50,51]. In fact, similarly, the homopolymer p(MPC) did not show any thermal transition in the investigated temperature range after the dehydration step.

Overall, the results suggest that, despite the random structure of the copolymers, the chemically incompatible polyphosphonate (or polyzwitterion or poly(ethylene glycol)) and polysiloxane side chains were able to self-assemble in separated microdomains characterized by the thermal behavior of the corresponding homopolymers. No sign of mixed phases was perceived, due to the minimal shift of the *T*_g_ values with respect to those of the corresponding homopolymer, even in those DSC curves where only one transition was detectable. Thus, the hydrophilic (PEtEP, MPC, PEGMA) and the hydrophobic (SiMA) side chains are supposedly immiscible in the solid state.

### 3.5. Hydrolisis of Poly(ethylphosphonate)-Based Polymers in Alkaline Conditions

**Figure 3:**
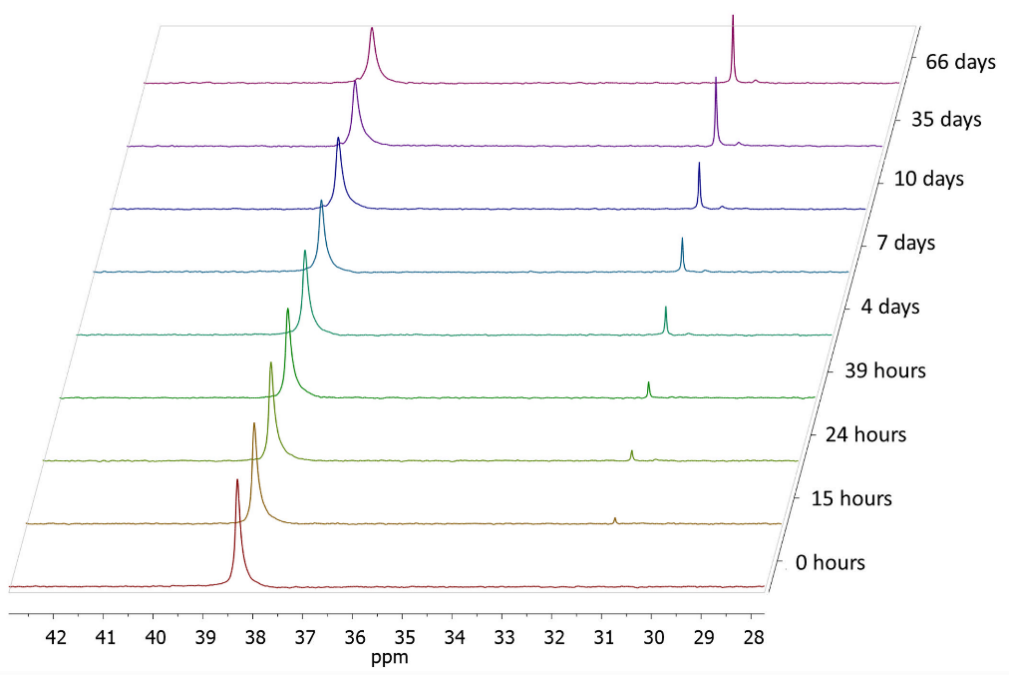
The ^31^P NMR spectra of p(PEtEPMA) in alkaline solution (pH ~ 10) at different times.

**Scheme 3:**
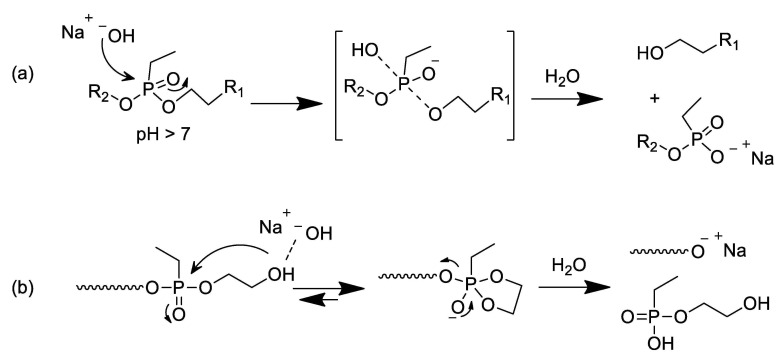
Proposed hydrolytic degradation mechanisms of poly(ethylene ethyl phosphonate)s in alkaline conditions: (**a**) nucleophilic attack of hydroxyl ions to the phosphorus center. (**b**) mechanism of backbiting reaction.

In order to evaluate the stability of the phosphonate side chains in alkaline conditions, PEtEP and p(PEtEP) were dissolved in a 50/50 *v*/*v* mixture of deuterated water and artificial seawater (pH ~ 8.2) or bicarbonate buffer (pH ~ 10). The hydrolysis reaction was followed by ^31^P NMR spectroscopy. The hydrolysis of the zwitterionic homopolymer p(MPC) was also monitored at a pH ~ 10. In the p(MPC) sample, a single resonance at −0.60 ppm was observed, which did not change even after 66 days at a pH ~ 10, indicating no degradation of the polymer under these conditions. In contrast, both the macromonomer PEtEPMA and homopolymer p(PEtEPMA) showed the formation of degradation products in the ^31^P NMR spectra (Figure 3). The phosphorus centers in the starting material exhibited a single resonance at 38.3 ppm. Already after 15 h at a pH ~ 10, a new resonance was detected upfield at ca. 31.1 ppm that gradually increased over time, which can be attributed to the main degradation product, i.e., ethyl ethylene phosphonate (a monoester). After prolonged degradation times, a second smaller signal was detectable at a lower chemical shift (30.6 ppm), which is probably attributed to ethyl phosphonic acid, i.e., after additional cleavage of the ester bond to ethylene glycol. From the literature, it is known that monoalkyl phosphonic acid esters and alkyl phosphonic acids (or their salts) can be detected in this spectral range [52,53]. The P–O bonds in polyphosphoesters are hydrolyzed under basic conditions [3,6,54], possibly via a nucleophilic attack of water or hydroxyl ions at the phosphorus center (Scheme 3a) or via a backbiting mechanism, which had been reported to be the major degradation pathway in polyphosphates with a terminal hydroxyethyl group [55] (Scheme 3b). Since the herein reported polymers did not carry a free hydroxyl group at their chain end, but were functionalized with a methacrylate group, one can suppose that first a chain scission needs to occur according to Scheme 3a, which is followed by a backbiting degradation [6]. In the further stages of the degradation, the remaining ester is also cleaved, revealing the free ethyl phosphonic acid.

The degree of hydrolysis (*HD*) was evaluated according to the following equation:

(1) $$HD\left(\%\right)=100\;\frac{Ap1+Ap2}{As+Ap1+Ap2}$$

where *Ap*1 and *Ap*2 are the integrated areas of the signals of the hydrolysis products at 31.1 ppm (ethyl ethylene phosphonate) and 30.6 ppm (ethyl phosphoric acid), respectively, and *As* is the area of the signal at 38.3 ppm of the initial polymer.

**Figure 4:**
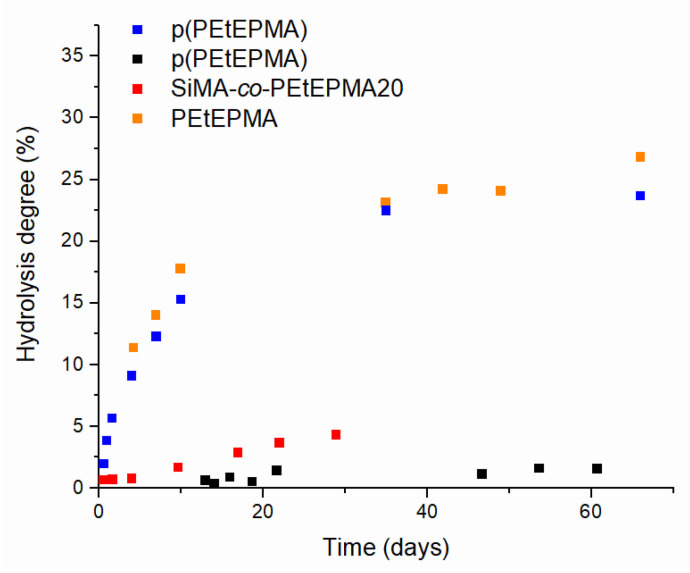
Hydrolysis kinetics of polyphosphonate-based samples in buffer solution (pH ~ 10) for p(PEtEPMA) (blue) and PEtEPMA (orange), in artificial sea water solution (pH ~ 8.2) for p(PEtEPMA) (black) and in THF-d8 with aliquot of buffer solution (pH ~ 10) for SiMA-*co*-PEtEP20 (red).

The hydrolysis kinetics of polyphosphonate-based (co)polymers is reported in Figure 4. Homopolymer p(PEtEPMA) and its monomer PEtEPMA presented similar hydrolysis kinetics at a pH ~ 10. In both cases, the degradation rate slowed down significantly after 20 days, and HD values seemed to reach a plateau at 20–30% after 35 days. Moreover, at a lower pH of ~ 8.2, typical of artificial sea water, the hydrolysis of p(PEtEP) was significantly slowed down. Hydrolysis was also observed for the copolymer SiMA-*co*-PEtEP20; however, as the polymer was water-insoluble, it was dissolved in deuterated THF with ~ 10 vol% of a bicarbonate buffer at a pH ~ 10. Despite the milder experimental conditions, the copolymer showed hydrolysis, even though the kinetics resulted to be significantly slower than that observed for the p(PEtEPMA) at a pH ~ 10. The lower hydrolysis rate was possibly also due to the higher hydrophobicity of the SiMA units in the copolymer. Similar findings had been reported for other non-water-soluble amphiphilic hydrolyzable copolymers, e.g., those containing silyl (meth)acrylate units [40]. Therefore, polyphosphonates were observed to undergo limited degradation (~2%) within two months at a pH ~ 8.2, while faster hydrolysis rates and more extensive degradation (20–30%) were observed at a pH ~ 10.

### 3.6. Preparation of Polymer Films

The non-water-soluble copolymers SiMA-*co*-PEtEP20, SiMA-*co*-MPC20, and SiMA-*co*-PEGMA28 were selected as surface additives to prepare PDMS-based three-layer films for biological assays, in order to compare the antifouling/fouling release performance of such different hydrophilic components. The selected copolymers possessed intentionally similar and relatively low mole percentages of the hydrophilic co-units, in order to avoid or limit possible leaching of the copolymer from the matrix when in contact with water. Moreover, the predominant SiMA component was anticipated to promote the chemical compatibility with the siloxane matrix.

The three-layer coatings consisted of a thin bottom layer (<5 μm thickness), a thicker middle layer (~400 μm thickness)—both composed of cross-linked PDMS—and a thin top layer of cross-linked PDMS loaded with 10 or 20 wt% surface-active copolymer (Figure 1). According to this procedure, the amphiphilic copolymer was physically dispersed, i.e., not chemically linked within the PDMS matrix in a semi-interpenetrating cross-linked network. For each layer, the cross-linking reaction occurred via a condensation sol–gel process at room temperature, that was catalysed by TBAF. The final cure was carried out at 120 °C for 12 h. While the thick middle layer provides the suitable elastomeric mechanical properties to the entire coating, the thin bottom layer (<5 μm thickness) ensured firm anchorage of the film to the glass surface, thus preventing a possible delamination phenomena of the coating during underwater evaluations. PDMS-based films similar to those reported here were proven to show a low Young modulus (*E* = 0.9 MPa) and high elongation at break (ε = 195%), consistent with the elastomeric nature of the PDMS-based coatings [20]. Finally, the physical dispersion of the amphiphilic copolymer in the top layer allowed for a concentration of the surface-active additive at the outer surface layers, closest to the film–water interface for more effective modulation of the surface properties. Thus, the three-layer geometry allowed the bulk/mechanical properties of the films to be independently controlled, basically due to the middle layer and its surface properties mainly depending on the nature of the amphiphilic copolymer.

### 3.7. Wettability and Surface Reconstruction

Water contact angles (*θ*) were measured in static sessile drop conditions after 50 s from the deposition of the drop, to evaluate the wettability of the film surface. *θ* values of the single-layer films of the pristine (co)polymers are collected in Table 3.

Homopolymers p(MPC) and p(PEtEPMA) were completely wettable, being *θ* ~ 0°. For these films, a partial solubilization of the polymer film in water was also noted, as expected.

All of the tested amphiphilic copolymers displayed a water contact angle in the range of 81–95°, indicating a general moderate hydrophobic nature of the film surface. In particular, SiMA-*co*-PEtEPMAx resulted to be the most hydrophilic copolymer class, followed by SiMA-*co*-PEGMA28. In any case, the *θ* values did not appear to be largely affected by the increasing amount of PEtEPMA co-units in the respective copolymers. One can speculate that the lower surface energy and the higher chain flexibility of the polysiloxane segments, with respect to polyphosphonate and even more to the zwitterion side chains, resulted in an efficient surface migration of such hydrophobic chains to the polymer–air interface that imparted an overall moderate hydrophobic character to the film surface. Surprisingly, despite the well-known high hydrophilicity of the zwitterion MPC, [50,56] SiMA-*co-*MPC20 films afforded the most hydrophobic surfaces with *θ* values of ~95°. In particular, for SiMA-*co-*MPC20, the migration of SiMA side chains to the surface might be predominant as a result of the ionic intra- and inter-macromolecular interactions among the zwitterion charged groups, thus limiting the migration of the hydrophilic side chains at the film surface.

The contact angles of the three-layer PDMS-based films were measured before (t = 0 days) and after immersion in deionized water for different times, up to 28 days (Table 4).

The measurements before immersion gave similar contact angles for all of the samples, including the PDMS control, confirming that the as-coated films did not expose a noticeable amount of hydrophilic co-units to air, even for copolymer loadings of 20 wt%.

All of the films, including PDMS, showed a progressive, slow decrease of *θ* values in the first 14 days of immersion. PDMS is known to undergo a process of surface reorganization after prolonged exposure to water to minimize the interfacial tension [57]. This is achieved by the progressive exposure of the polar Si–O–Si groups to the water–polymer interface, and reorienting the methyl groups into the bulk of the polymer film. After 21 days of immersion, all of the amphiphilic film surfaces, except SiMA-*co*-PEGMA28_20, showed a significant decrease in the water contact angle of 9–25° with respect to unmodified PDMS. In particular, this phenomenon was more marked for SiMA-*co*-PEtEP20_z-based films, which reached values as low as 65° in the case of SiMA-*co*-PEtEP20_10 films after immersion for 28 days. Moreover, a trend in the decrease of surface reconstruction kinetics was found in passing from PEtEP to MPC and to PEG. The continued and progressive hydrophilization of the amphiphilic PDMS-based films in general, and of SiMA-*co*-PEtEP20_z in particular, is attributed to the effective surface migration and major exposure of the hydrophilic co-units of the additive copolymer to the polymer–water interface.

### 3.8. Biological Test with Marine Organisms

#### 3.8.1. Ficopomatus Enigmaticus Settlement and Detachment Assay

**Figure 5:**
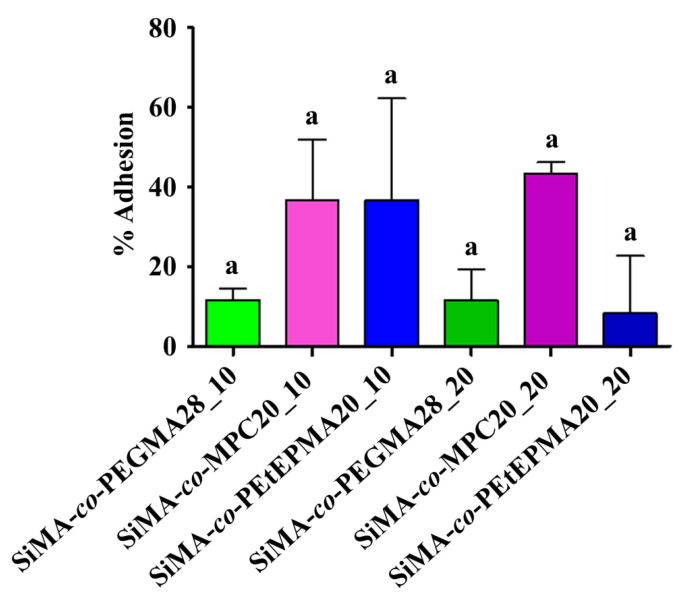
Mean percentage of adhesion of *F. enigmaticus* competent larvae on different surface samples, after five days of exposure. An ANOVA analysis (n = 6) followed by a Tukey post-hoc test were performed for multiple comparisons. Not-shared letters indicate statistically significant differences, *p* ≤ 0.05. Adhesion percentage for PDMS was 16.67 ± 6.83% (mean ± standard deviation).

**Figure 6:**
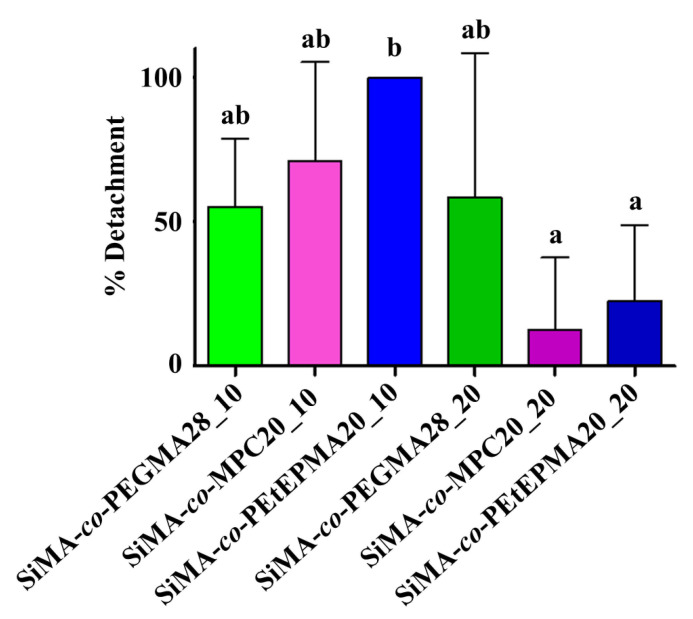
Percentage of detachment of *F. enigmaticus* adults from different surface samples, after 25 Pa shear stress exposure (15 min). An ANOVA analysis (n = 3) followed by a Tukey post-hoc test were performed for multiple comparisons. Not-shared letters indicate statistically significant differences, *p* ≤ 0.05. Detachment percentage for PDMS was 70.84 ± 20.97% (mean ± standard deviation).

The antifouling performance of three-layer films was assessed against *F. enigmaticus* on a laboratory scale. In the settlement (adhesion) test, competent larvae of *F. enigmaticus* were directly pipetted on the polymer surfaces, and the percentage of adhesion was evaluated after five days of incubation. Figure 5 shows that the percentage of adhered larvae on amphiphilic films was lower than ~40% in any case. The initial hydrophobic surface was beneficial in slowing the settlement of the larvae, which are known to better adhere to hydrophilic rather than hydrophobic substrates [20]. Overall, the different copolymer chemistries and loadings did not show clear trends and statistically significant differences, thus indicating that the PEGMA, MPC, and PEtEP hydrophilic components possess a similar antifouling performance against this specific organism. Nevertheless, it is known from the literature that a marked hydrophilic nature of the coatings promotes the release of *F. enigmaticus* under relatively low shear stresses [58]. Consistently, the detachment percentage of *F. enigmaticus* (Figure 6) was found to be the highest for SiMA-*co*-PEtEPMA20_10, which displayed the most hydrophilic surface, wherein its water contact angle after 28 days of immersion in water was the lowest. Moreover, *F. enigmaticus* detachment was generally easier from the films with a lower wt% of copolymer additive, although this trend was not statistically significant.

#### 3.8.2. Navicula Salinicola Settlement and Detachment Assay

**Figure 7:**
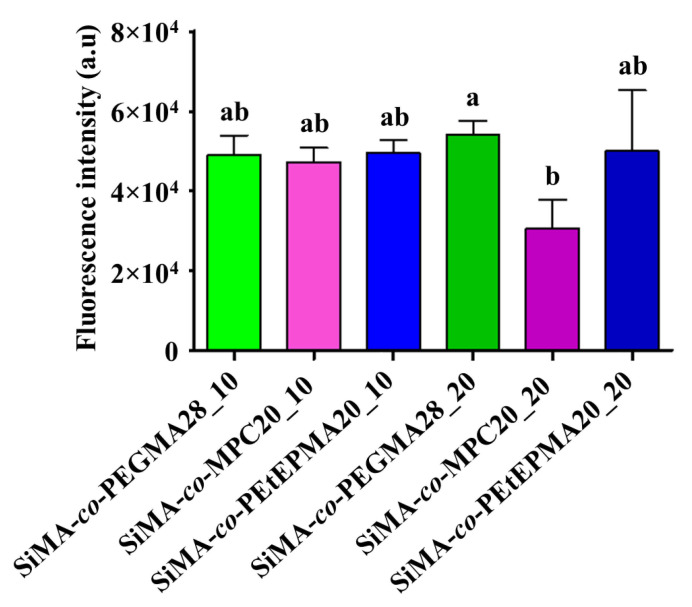
Intensity of fluorescence emitted by *N. salinicola* cells adherent to different surface samples, after 24 h of exposure. An ANOVA analysis (n = 3) followed by a Tukey post-hoc test were performed for multiple comparisons. Not-shared letters indicate statistically significant differences, *p* ≤ 0.05. Fluorescence intensity for was 5.38 × 10^4^ ± 0.52 × 10^4^ a.u. (mean ± standard deviation).

**Figure 8:**
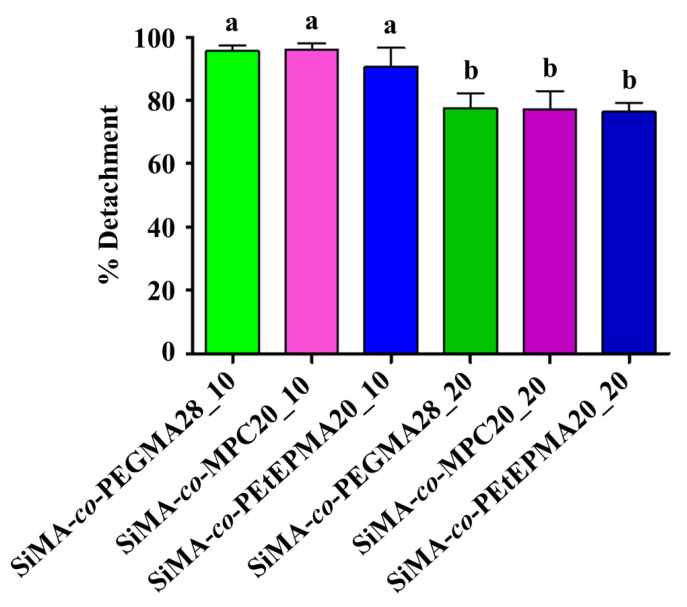
Percentage of detachment of *N. salinicola* cells from different surface samples, after 5 Pa shear stress exposure (five minutes). An ANOVA analysis (n = 3) followed by a Tukey post-hoc test were performed for multiple comparisons. Not-shared letters indicate statistically significant differences, *p* ≤ 0.05. Detachment percentage for PDMS was 93.50 ± 5.97% (mean ± standard deviation).

Amphiphilic films were also subjected to laboratory assays to evaluate the colonization and removal of the diatom *N. salinicola*. The settlement was quite similar on all of the tested surfaces, being significantly lower only on SiMA-*co*-MPC20_20 (Figure 7). Thus, the presence of phosphorylcholine side chains in the copolymer additive improved the antifouling properties of the coatings against diatom biofilm. Figure 8 shows the percentage of diatom removal after 5 min of exposure to a low wall shear stress of 5 Pa. For all of the tested surfaces, the mean cell removal percentage was higher than ~ 75% even for the worst performing coating. In general, the chemical nature of the hydrophilic co-units in the copolymer appeared to not significantly affect the detachment of the tested diatom, with PEtEPMA performing as well as PEGMA and MPC. In agreement with what was observed for the detachment of *F. enigmaticus*, the removal percentage of *N. salinicola* was higher for films with the lower amount of copolymer loading, although the differences were not statistically significant.

## 4. Conclusions

Amphiphilic methacrylic graft copolymers, composed of the hydrophobic polysiloxane (SiMA) side chain and three different hydrophilic pendant chains—polyphosphonate (PEtEPMA), polyzwitterion (MPC), and polyethylene glycol (PEGMA)—were synthesized and used as surface-active copolymers in two different loadings (10 and 20 wt%) in a condensation cure PDMS matrix. PEtEPMA-based films derived therefrom were proven to be more susceptible to surface reconstruction after prolonged exposure to water than the corresponding MPC and PEGMA-based coatings. In particular, the extent of the decrease in the water contact angle (*θ*) increased in going from PEGMA to MPC up to PEtEPMA, for which the reduction in *θ* was as high as ~40°.

Biological assays against two different model marine organisms revealed that the settlement of *F. enigmaticus* larvae was generally low (≤40%) on all of the tested films after five days of incubation. The complete removal of adults was achieved for SiMA-*co*-PEtEPMA20_10, which displayed the most hydrophilic surface upon immersion in water. The biological performance against *N. salinicola* was comparable to those of the MPC- and PEGMA-based films. However, for both of the tested organisms, a trend was observed, suggesting that removal was generally higher for films containing the lower amount of copolymer.

The present study is the first to use polyphosphonate-based copolymers for marine antifouling applications. Further, we have compared the wettability and AF/FR properties of films containing polyphosphonate, polyzwitterions, and PEG. Overall, polyphosphonates were identified as a potential alternative to polyzwitterionic and PEGylated antifouling coatings. In addition, as polyphosphonates can be prepared with different hydrolysis kinetics, this can add synergistically to their amphiphilicity in producing evolving, environmentally-responsive, and erodible surfaces effective in combating marine fouling.

## Figures and Tables

**Table 1:** Polymerization conditions and chemical-physical properties of SiMA-*co*-PEtEPx copolymers.

Copolymer	PEtEP/MPC/PEGMA			*p* ^d^	Yield	Water Solubility
	(mol%) ^a^	(mol%) ^b^	(wt%) ^c^	(%)	(%)	
p(SiMA)	0	0	0	96	74	no
p(PEtEPMA)	100	100	100	76	32	complete
SiMA-*co*-PEtEPMA20	25	20	44	94	66	no
SiMA-*co*-PEtEPMA53	50	53	78	96	44	up to 1 g/L
SiMA-*co*-PEtEPMA66	70	66	86	94	54	up to 1 g/L
SiMA-*co*-MPC20	30	20	7	96	49	no
SiMA-*co*-PEGMA28	25	28	10	85	62	no

^a^ Mole percentage in the feed. ^b^ Mole percentage of the hydrophilic units in the copolymer evaluated by ^1^H NMR. ^c^ Weight percentage of the hydrophilic units in the copolymer. ^d^ Monomer conversion.

**Table 2:** DSC data for the amphiphilic copolymers (taken from the second heating cycle at 10 K/min).

Copolymer	*T*_g_^1 b^ (°C)	∆*C*_p_^1 b^ (J/g K)	*T*_g_^2 c^ (°C)	∆*C*_p_^2 c^ (J/g K)
p(SiMA) ^a^	−124	1.160	-	-
p(MPC)	-	-	-	-
p(PEtEPMA)	-	-	−31	0.54
PEtEPMA	-	-	−31	0.46
SiMA-*co*-PEtEPMA20	−121	0.26	−29	0.27
SiMA-*co*-PEtEPMA53	-	-	−27	0.40
SiMA-*co*-PEtEPMA66	-	-	−26	0.40
SiMA-*co*-MPC20	−119	0.49	-	-
SiMA-*co*-PEGMA28	−119	0.47	-	-

^a^ Data from literature ^b^ Glass transition temperature and specific heat change associated with SiMA enriched domains. ^c^ Glass transition temperature and specific heat change associated with PEtEPMA/MPC/PEGMA units.

**Table 3:** Water contact angles of pristine (co)polymer films ^a^.

Polymer Films	*θ* (°)
p(MPC)	~0
p(PEtEPMA)	~0
SiMA-*co*-PEtEPMA20	81 ± 3
SiMA-*co*-PEtEPMA53	87 ± 4
SiMA-*co*-PEtEPMA66	85 ± 6
SiMA-*co*-MPC20	95 ± 2
SiMA-*co*-PEGMA28	89 ± 4

^a^ Films were prepared according to a single-layer geometry.

**Table 4:** Water contact angles of the three-layer PDMS-based films.

PDMS-Based Films	0 Days *θ* (°)	7 Days *θ* (°)	14 Days *θ* (°)	21 Days *θ* (°)	28 Days *θ* (°)
SiMA-*co*-PEGMA28_10	101 ± 1	98 ± 3	96 ± 2	88 ± 4	74 ± 3
SiMA-*co*-PEGMA28_20	103 ± 1	97 ± 1	97 ± 2	93 ± 6	96 ± 5
SiMA-*co*-MPC20_10	107 ± 3	96 ± 2	98 ± 2	88 ± 4	85 ± 5
SiMA-*co*-MPC20_20	108 ± 4	95 ± 1	94 ± 3	86 ± 3	68 ± 3
SiMA-*co*-PEtEPMA20_10	104 ± 3	95 ± 3	93 ± 4	72 ± 5	65 ± 2
SiMA-*co*-PEtEPMA20_20	103 ± 2	96 ± 2	94 ± 4	75 ± 3	70 ± 4
PDMS	102 ± 1	100 ± 2	99 ± 2	97 ± 1	94 ± 1

## Data Availability

The data presented in this study are available on request from the corresponding author.
